# Hyaluronic Acid Treatment of Post-Extraction Tooth Socket Healing in Subjects with Diabetes Mellitus Type 2: A Randomized Split-Mouth Controlled Study

**DOI:** 10.3390/jcm13020452

**Published:** 2024-01-14

**Authors:** Tiziana Ruggiero, Massimo Carossa, Davide Camisassa, Marta Bezzi, Giulia Rivetti, Vincenzo Nobile, Renato Pol

**Affiliations:** 1 C.I.R. Dental School, Department of Surgical Sciences, University of Turin, 10126 Turin, Italy; 2 Politecnico di Torino, Corso Duca degli Abruzzi 24, 10129 Turin, Italy; 3 Complife Italia S.r.l., Via Mons. Angelini 21, 27028 San Martino Siccomario, Italy

**Keywords:** diabetes mellitus, hyaluronic acid, tooth socket, healing, post-extractive

## Abstract

The present study aimed to investigate the effect of HA in improving post-extraction socket healing in subjects with diabetes mellitus (DM) type 2. DM patients requiring bilateral extraction of the homologous teeth were visited at the C.I.R. Dental School, University of Turin. After the extractions, one site was randomly assigned to the test (T) group (postoperative application of HA), while the other site was assigned to the control group (C, no treatment). Patients were then followed after 3, 7, 14, and 21 days. Primary outcomes were the healing index and socket closure. The Mann-Whitney test or the Student’s t-test was used for nonparametric or parametric distributed variables. The chi-square test was used if the estimated data in any given cell were >5, otherwise the Fisher test was adopted. A *p* < 0.05 was considered statistically significant. In total, 36 patients (*n* = 36) were enrolled in this study for a total of 72 extractions (*n* = 72). Sockets treated with HA showed significantly (*p* < 0.05) better healing index values at day 7 (*p* = 0.01) and at day 14 (*p* = 0.02) and significantly (*p* < 0.05) better socket closure values at day 3 (*p* = 0.04), day 7 (*p* = 0.001) and day 14 (*p* = 0.001) compared to the C group. Based on the clinical results, HA seems to be promising in improving the timing and the quality of post-extractive wound healing in DM patients. Further clinical research, as well as histological investigations, are required to confirm the results.

## 1. Introduction

Diabetes mellitus (DM) is a systemic metabolic condition that causes hyperglycemia and microvascular consequences as a result of impaired insulin and faulty insulin secretion [1]. It can be divided into DM type 1 and type 2. Among them, DM type 2 is reported to represent 90% of the diagnosed cases [2]. In the past three decades, the prevalence of type 2 diabetes has risen dramatically in countries of all income levels [3]. As highlighted by Nazir et al. [4], more than 90% of diabetic patients present with oral manifestations, making it the third most common chronic disease among the population of dental patients [5]. As extensively investigated and described in a recent review on the topic by Yang et al. [6], one of the critical aspects of DM patients undergoing dental procedures is the delay in wound healing after tooth extractions. The cause for slow wound healing in DM patients is complex and involves the altered expression of all the cells normally involved in the healing, as well as a dysregulation in the production of growth factors and cytokines [6]. As a result, the postextractive socket of diabetic patients requires more time to heal compared to healthy patients, increasing the risk of infections and the likelihood of taking more drugs and antibiotics. Generally, the difference in healing time between DM and non-DM patients is likely evident 7 days after the extraction, as highlighted in the study by Gadicherla et al. [7]. Indeed, DM patients have more complications and a longer period of post-extraction wound healing than healthy patients, followed by unfavorable post-extraction alveolus changes [8,9,10]. Despite proper radiological diagnosis and proper surgical and prosthetic treatment plans, due to bone alteration, DM patients additionally suffer from impaired osseointegration, elevated risk of peri-implantitis, and higher levels of implant failure [11]. Commonly, different materials and strategies are adopted to better seal the postextractive site [12,13,14]. Regarding DM patients, different procedures have been proposed over the years, including the use of growth factors [15,16], synthetic drugs [17,18], and laser therapy [19]. However, despite positive benefits, the results are sometimes controversial, and, therefore, research on the topic remains open.

In recent years, hyaluronic acid (HA), a naturally occurring glycosaminoglycan, has gained attention due to its properties in improving cell migration and proliferation [20,21]. In regard to oral tissues, it was previously described [21] to act by enhancing and maintaining the oral fibroblast proliferation and activities and to enhance the expression of genes typically involved in wound healing, such as type III collagen and growth factor-β3 [21]. To date, different studies have investigated the usage of HA for dentistry applications [22,23]. In an in vitro study on periodontal ligament cells, Fujioka-Kobayashi et al. [20] reported the positive effect of HA in enhancing and maintaining cell proliferation, viability, and osteogenic differentiation. In agreement, from a biological standpoint, the in vitro study by Asparuhova et al. [21] highlighted how HA formulations are capable of enhancing the migration and proliferation of cell types typically involved in soft tissue wound healing. In vivo studies also demonstrated the beneficial effect of HA in soft tissue healing. Gocmen et al. [24] found HA to facilitate angiogenesis and provide anti-inflammatory effects after third molar extraction, while Alcântara et al. [25], in a triple-blind randomized controlled trial, found a positive effect of HA in bone remodeling and repair in dental sockets. Furthermore, a recent systematic review by Maria de Souza et al. [26] on the topic highlighted a correlation between the application of HA and a reduction of postoperative pain. Moreover, HA is widely used in other branches of medicine, and neither contraindications nor interactions with drugs have been reported [27,28,29,30]. However, the investigation of the possible benefits of applying HA in the postextractive sockets of diabetic patients is currently lacking.

Therefore, the aim of the present study is to investigate the effect of HA in improving post-extraction tooth socket healing in subjects with DM type 2.

The null hypothesis is that HA can significantly improve the postextractive healing of diabetic patients compared to no treatment.

## 2. Materials and Methods

All the study procedures were carried out according to the World Medical Association’s (WMA) Helsinki Declaration and its amendments (Ethical Principles for Medical Research Involving Human Subjects, adopted by the 18th WMA General Assembly Helsinki, Finland, June 1964, and amendments). All patients enrolled in the study were thoroughly informed about the research purpose and signed an informed consent form prior to undergoing the procedures. The present study was reported following the CONSORT 2010 guidelines [31]. The study protocol and the research were approved by the local ethical committee of the University of Turin (approval code 0100924 on 15 September 2022). The trial was retrospectively registered at ClinicalTrials.gov (ID: NCT05896319, Registration date: 9 June 2023).

### 2.1. Study Design

The present study was designed as a single-center split-mouth randomized controlled trial. A split-mouth design was used to reduce the possibility of bias caused by potential variations in uncontrolled variables between patients. Patients requiring bilateral extraction of the homologous, not impacted, teeth were visited at the C.I.R. (Interdepartmental Research Center) Dental School, Section of Oral Surgery, Department of Surgical Sciences, University of Turin from September 2022 to February 2023. The inclusion criteria were the following: age ≥ 18 years old; diabetic type 2 patients with a positive history of diabetes complications (e.g., nephropathy, neuropathy, retinopathy, cardiopathy, peripheral vascular disease); requirement of bilateral extractions of the homologous, not impacted, teeth; consent for enrollment in this study; and availability to attend the control visit. The exclusion criteria were the following: the presence of platelet dysfunction; presence of thrombocytopenia; corticosteroid treatment; smokers; refusal to participate in this study; uncontrolled diabetes; assumption of drugs possibly interacting with the wound healing; extractions requiring the elevation of a flap; teeth requiring separation with burs; ankylosed teeth requiring the usage of a burs to allow the extraction; and apical fractures during extractions.

After the extractions were carried out in the same appointment, following the split-mouth design of this study, one site was randomly assigned through a computer-generated random sequence of numbers (SPSS 24.0; SPSS Inc., Chicago, IL, USA) to the test (T) group, while the other one was assigned to the control group (C).

The T group (treated) included the postoperative application of HA (aminogam^®^, PROFESSIONAL DIETETICS S.p.A., Via Ciro Menotti, 1/A, 20129, Milan, Italy) 3 times per day (8 h distance between each application) for 7 days after oral hygiene and without swallowing, eating, or drinking for one hour after the application, as follows: “wash your hands thoroughly before each application, apply a layer of gel on the injured socket until it is fully covered, massage with a finger in order to facilitate spreading of the product over the treated area and compressing the product with gauze”.

In detail, the product adopted is composed of HA and synthetic amino acids that serve as the precursors of collagen. The product qualitative ingredients list was as follows: purified water, sodium hyaluronate, glycine, L-proline, L-Leucine, L-lysine HCl, methyl parahydroxybenzoate, propyl parahydroxybenzoate, sorbitol, polyvinylpyrrolidone, sodium hydroxide, propylene glycol, and tetrasodium EDTA.

C group (untreated) included: no treatment.

All the surgeries were performed by the same experienced clinician who specialized in oral surgery (R.P.) and was blinded to the group allocation of the sites. All the pre- and postoperative assessments were performed by two calibrated and trained operators (T.R. and G.R.) who were blinded to the T and C group allocation. Cohen’s kappa statistic was adopted to calculate observer agreement.

### 2.2. Preoperative Assessment

Prior to extractions, patients underwent a professional oral hygiene session while the dentist clinically and radiographically evaluated the following items:

- Demographic characteristics of the subjects enrolled in this study. The following data was collected: gender; age; ethnic origin; body mass index (BMI); smoking habits.
- Diabetes-related data. The following data were collected: duration of diabetes; diabetes status (blood sugar level) evaluated on the day of the surgery; glycated hemoglobin (HbA1c) level; end-organ disease score.
- Pre-operative status of the teeth that required extraction. The following information was collected: single or multirooted teeth; the presence of cavities; pulp vitality and previously endodontic treatments; the presence of peri-apical lesion;
- The degree of difficulty of the extraction [32,33] (Table 1). The operative difficulty was classified according to 3 degrees:
  - Low: all low-grade parameters, no more than one intermediate-grade parameter;
  - Intermediate: more than one parameter of intermediate difficulty, no parameter of high difficulty;
  - High: one or more high-grade parameters.

**Table 1:** Evaluation grid of the pre-operative difficulty level and parameters considered.

Parameters	Low Difficulty	Medium Difficulty	High Difficulty
**Patient collaboration**	Cooperative	Suspicious	Uncooperative
**Space**	Higher than the MD crown size	Equal to the MD crown size	Smaller than the MD crown size
**Crown integrity**	Intact crown	Incomplete crown	Crown absent
**Root anatomy**	Low difficulty	Medium diffulty	High difficulty

Patient’s systemic risk (Table 2). A model derived from a study by Aronovich et al. [34] in which the relationship between the degree of glycemic control and the results following tooth extraction were evaluated, bearing in mind the diagnosis and management of the diabetic patient described by Mozzati and Pol [15].

All the above parameters were acquired in order to have a comparison for inter- and intrapatients to avoid any bias related to different T0 parameters.

### 2.3. Surgical Appointments

The homolog bilateral extractions were carried out in the same appointment. Local anesthesia (plexus or alveolar nerve block infiltration) was given using 1.8 mL vials of 3% mepivacaine without vasoconstrictor (Opticain, Molteni Dental Srl, Firenze, Italy). All the extractions were performed in a nontraumatic way and without a full-thickness mucoperiosteal flap elevation to preserve the bone crest and the soft tissue integrity. After the extractions, the sockets were cleansed (removal of infected tissue) to help wound healing. If the patient required sutures for blood dyscrasias, this was applied on both the sites (nonabsorbable silk suture, stainless steel Permahand 3/0, Ethicon, Sommerville, NJ, USA). The sockets were then compressed with a sterile gauze. Patients were then instructed with postoperative recommendations, including hygienic instructions and a tube (15 mL) of the test product. Since anti-inflammatories act by inhibiting the production of cytokines and the inflammatory response, and they can consequently alter the mechanism of action of HA as well as the perception of pain, no anti-inflammatory drugs or antibiotics were prescribed following the extraction. The need to administer the antibiotic after the extraction was considered a negative postoperative evaluation parameter, as it was a sign of complication due to infection.

### 2.4. Clinical Outcomes

**Figure 1:**
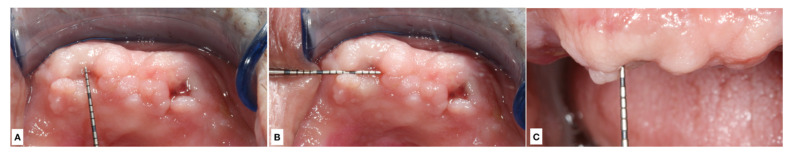
Images showing the calculation of the measurements (millimeters) of the maximum OV diameter (**A**), the maximum MD diameter (**B**), and the maximum SD (**C**).

Primary clinical outcomes were:

- Healing index [35]. To evaluate the healing of the postextractive sockets, a simplified version of Landry’s index [35] was adopted, considering only 3 possible scores for each of the 4 parameters considered: tissue color (1 = 100% pink gum; 2 = <50% hyperemic gum; 3 = >50% hyperemic gingiva), bleeding (1 = absent; 2 = provoked by palpation; 3 = spontaneous), granulation tissue (1 = pink and firm; 2 = red and soft; 3 = brittle), suppuration (1 = no accumulation of plaque on the margins; 2 = evident plaque on the margins; 3 = suppuration/alveolitis). In this index, a score of 4 corresponds to excellent healing; conversely, a score of 12 corresponds to poor healing.
- Socket closure. It was defined as the ratio between the volume of the healing socket at a given time (3, 7, 14, 21 days) and the volume of the socket at T0. It was calculated by measuring (millimeters) the maximum oral vestibule (OV) diameter (Figure 1A), the maximum mesiodistal (MD) diameter (Figure 1B), and the maximum socket depth (SD) (Figure 1C).

MD diameter was measured at the point of the maximum MD width of the socket for both single-rooted and multi-rooted teeth. OV diameter was measured at the point of the maximum oral vestibule width of the socket or of each root (considering only the maximum value for the plurirooted teeth). SD was measured as the distance between the gingival margin and the socket bone at the point of its maximum depth (without forcing the probe). After training of the examiners (T.R. and G.R.), all the measurements were performed using a HuFriedy PCPUNC 15 probe (HuFriedy, Chicago, IL, USA) on the day of the surgery (D0), and after 3 (D3), 7 (D7), and 14 (D14) days from the surgery, memorizing the reference points for each patient to avoid possible measurements errors. An additional follow-up visit after 21 (D21) days was planned in case the socket was not completely healed after 21 days from the surgery.

Secondary outcomes were:

Pain measurement on a Visual Analogue Scale (VAS); the need to add a follow-up visit after 21 days from the surgery; the need to prescribe the use of antibiotics to counteract postoperative symptoms; overinfection of the granulation tissue; the need for reoperation in the case of complications during the healing process; a self-assessment questionnaire that included the following questions: what site healed most comfortably? Would you prefer the application of the product for future extractions (after revealing what site was treated with the product)? Did you notice more bleeding in either of the two sites? At what site did you experience more pain?

### 2.5. Sample Size Calculation

A total of 20 patients and 40 extractions were calculated as required for this study based on a previously published article [36]. However, based on the usual large flow of patients arriving at the Section of Oral Surgery, Department of Surgical Sciences, University of Turin, a minimum sample size of 35 patients and 70 extractions was calculated to increase the study power.

### 2.6. Statistical Analysis

Continuous variables are reported as mean ± standard deviation (sd). The Mann-Whitney test or the Student’s *t*-test were used for nonparametric or parametric distributed variables, respectively. Categorical variables (dichotomous or polychotomous) were reported as counts and/or percentages. The statistical analysis of categorical variables was carried out on contingency tables or RxC cross-correlation matrices. The chi-square test (with Yates correction for 2 × 2 tables) was used if the estimated data in any given cell were >5; otherwise, the Fisher test was used. The Risk Ratio (RR) or the Odds Ratio (OR) was used in the case of 2 × 2 tables. A *p* < 0.05 was considered statistically significant. For the RR calculation, the 95% confidence interval excluded 1.

## 3. Results

In total, 36 patients (*n* = 36), 17 males (47%) and 19 females (53%) with a mean age of 67.28 ± 11.22, met the inclusion criteria and were enrolled in this study for a total extraction volume of 72 teeth (*n* = 72). T group and C group comprised 36 sites each (*n* = 36 per group). Excellent intraobserver (kappa values of 0.78 and 0.80) and interobserver (a kappa value of 0.80) agreements were recorded in this study.

Demographic and baseline characteristics are reported in Table 3.

No drop-outs or patients lost to follow-up were recorded. All subjects were included in the efficacy and safety analysis data set. The tested product was well tolerated. No adverse reactions occurred during the study period. No requirement of antibiotic prescription was recorded for any of the enrolled patients.

No differences were observed in the patient collaboration. Only one subject was classified as suspicious.

Table 4 shows the baseline pre-operative data recorded regarding teeth status and surgical difficulty between the T and C groups.

Based on the results, no statistically significant differences (*p* > 0.05) were highlighted between the two groups for any of the considered baseline variables. Therefore, it is possible to conclude that both the untreated and the treated sites were similar, indicating an unbiased randomization and absence of covariates.

### 3.1. Healing Index

Table 5 shows data related to the healing index recorded at different time points.

Sockets treated with HA (T group) showed significantly (*p* < 0.05) better healing index values at D7 (*p* = 0.01) and D14 (*p* = 0.02), while no statistically significant differences were highlighted at D3 (*p* = 0.08) and D21 (*p* = 1).

In regard to the % of sockets that presented with optimal healing (healing index = 4), a statistically significant difference (*p* < 0.05) was highlighted at D14 (*p* = 0.004), with sockets treated with HA showing the better result. At the last follow-up (D21), all the sockets, regardless of the group (*p* = 1), reached an excellent healing score.

### 3.2. OV, MD, and SD

Values recorded in regard to OV diameters showed a statistically significant difference (*p* = 0.03) in favor of the T group at D3, while no statistically significant difference (*p* > 0.05) was recorded between the two groups at the other follow-up times. Regarding the MD diameters, a statistically significant difference was highlighted at D3 (*p* = 0.03) in favor of sites treated with HA (T group), while no statistically significant difference (*p* > 0.05) was recorded between the two groups at the other follow-up times. In regard to SD, a statistically significant difference was recorded at D14 (*p* = 0.04) in favor of the T group, while no statistically significant difference (*p* > 0.05) was recorded between the two groups at the other follow-up times. At the last follow-up (D21), no statistically significant difference was highlighted between the groups for any of the three parameters considered.

### 3.3. Socket Closure

No statistically significant difference (*p* = 30) was recorded between the two groups at D0; therefore, the initial conditions were superimposable.

Table 6 shows values recorded regarding socket closure.

Sockets treated with HA (T group) showed significantly (*p* < 0.05) better socket closure values at D3 (*p* = 0.04), D7 (*p* = 0.001), and D14 (*p* = 0.001) compared to the C group.

Regarding the % of sockets fully closed, no statistically significant difference (*p* > 0.05) was highlighted between the two groups at any time point. At the last follow-up (D21), all the sockets, regardless of the group (*p* = 1), reached the perfect score.

### 3.4. VAS

Table 7 shows data recorded in regard to VAS at different time points.

Data recorded were statistically (*p* < 0.05) more favorable for the T Group at D3, D7, and D14. At the last follow-up (D21), the same VAS scores were recorded for both of the groups (*p* = 0.9999999).

### 3.5. Questionnaire

An analysis of the patient questionnaires demonstrated that all patients (100%) found healing with HA more comfortable and would prefer the product for future extractions. A total of 89% of patients noted more bleeding at the control site, and 56% reported more pain in the socket not treated with HA.

## 4. Discussion

The aim of the present split-mouth randomized control trial was to investigate whether the employment of HA gel can provide benefits in post-extraction tooth socket healing in subjects with DM type 2. This type of systemic patient often suffers from delayed wound healing and unfavorable changes in the tridimensional remodeling of the socket [4,10]. Therefore, research is continuing to focus on how to improve the postextractive wound healing of these patients, both from a quality and timely standpoint. The present study was designed as a split-mouth study, where one site was randomly assigned to the T group (application of HA gel), while the other site in the same patient was assigned to the C group (no treatment). A total of 36 patients were enrolled for a total of 72 teeth extractions (*n* = 36 extractions per group). Only patients with a prior positive history of diabetes complications were considered. This inclusion criteria was chosen to test the product on patients that, based on their history, may be more likely to present with diabetes-related complications. Since statistically significant differences were highlighted between the two groups, the null hypothesis was accepted.

The first primary outcome of this study was the healing index. In order to clinically evaluate the healing of the soft tissues following extraction, the Landry index [35] was used, which was modified so that it could be applied to the evaluation of the post-extraction socket. In fact, this index was devised by the authors to evaluate the healing of soft tissues following periodontal surgery, with suturing and closure of the wound by primary intention. Since the healing of the post-extraction socket recognizes a healing mechanism by secondary intention, and in order to be able to evaluate the regeneration of the soft tissues, some of the parameters obtained from the original index were adopted in this study, modifying them so as to be able to apply them to healing by second intention. Indeed, the presence of granulation tissue between the edges of the wound, i.e., between the walls of the alveolus, is considered a positive parameter, as it lays the foundations for the formation of the connective tissue, while the previous authors considered it a negative factor in wound closure for primary intention.

In regard to the healing index, a statistically better result was highlighted for the HA group at D7 and D14, while no statistically significant difference was found at D3 and D21. This result may indicate how the application of HA may play a significant role in improving postextractive wound healing in the first 2 weeks postsurgery. These timeframes are moments in which the diabetic patient has a greater risk of superinfection [4,6], and, therefore, it is fundamental to have a fast and predictable wound-healing process. In the present study, no requirement for antibiotic prescription was recorded for any of the treated patients. The topic related to the requirement of routinely prescribing antibiotics to DM patients prior to tooth extraction is currently controversial, and, as highlighted in recently published articles on the topic, a final validated protocol is currently absent [37,38,39]. The possible role of HA gels in improving healing was also highlighted by analyzing the % of ‘‘excellent healing’’ (healing index = 4) per group. A statistically significant difference between the two groups was highlighted at D7 and D14, with 97.2% (*n* = 35) of sites for the T group and 72.2% (*n* = 26) of sites for the C group that reached excellent healing at D14. This result may indicate that sites not treated are subject to delayed complete healing of the wound, confirming what is currently known about wound healing in diabetic patients. Indeed, only 1 patient of T group, while 10 patients of group C, required 21 days to reach a healing index of 4.

The second primary outcome that was analyzed was the socket closure. Based on the results, a statistically significant difference (*p* < 0.05) was highlighted between the groups at D3, D7, and D14, with the T group presenting a better remodeling of the socket compared to the C group. On the contrary, when considering the % of fully closed sockets, no statistically significant difference (*p* > 0.05) was highlighted between the two groups at any time point. This result may indicate that, despite the full closure of the sockets visually observed, the quality and timing of the healing could be significantly influenced by the application of the HA gel.

As secondary outcomes, the VAS scale and patient questionnaires were analyzed. For both of the outcomes, positive results in favor of the HA gels were found. All of the patients (100%) found healing with HA more comfortable and would prefer the product for future extractions. A total of 89% of patients noted more bleeding at the control site, and 56% reported more pain in the socket not treated with HA.

Following this result, the application of HA gels seems to be promising in improving the timing, quality, and patients’ experience during the wound healing process.

However, some considerations must be taken into account. The whole number of sockets reach the perfect scores, both considering the healing index and the socket closure, after 21 days from the surgery, regardless of the application or not of the HA gel. No complications or superinfections were noted for either of the two groups. This result may indicate how the application of HA may represent a way to speed up the healing process and lower the risk of infection by decreasing the timing of wound closure; however, the final healing observed in the longest follow-up period (D21) was not influenced by the application of the HA gel. Furthermore, it must be noted how the study design allowed evaluation only of clinical outcomes, while laboratory analysis, such as histological analysis, was not performed to biologically evaluate the healing of the two groups. No data about bone remodeling were collected. This is a limitation inherent to the short follow-up period applied in this study. Further studies with higher follow-up periods are necessary to evaluate whether the HA gel may improve bone healing and socket remodeling after teeth extraction. Indeed, socket preservation is sometimes challenging even in healthy patients [12,40], and it becomes more important for diabetic patients who seek to undergo implant therapy in the future. Additional limitations are represented by the small sample size. Further studies with larger sample sizes are required to confirm the results of the present study. Furthermore, only extractions that did not require the elevation of a flap nor the usage of a bur to allow the extractions were considered. Further studies are required, and it would be interesting to test the effects of the products in more complex extractions in this type of systemic patient.

The results of the present study are in agreement with the randomized control trial by Marin et al. [41], who followed 30 diabetic patients treated with and without HA up to 25 days post-extractions. The authors’ findings showed statistically better healing for the sockets treated with HA after 10 and 15 days and significantly increased socket closure for all the time points considered. Contrary to the present study, the socket closure was statistically higher for the HA group compared to the control group, even 25 days post-extraction. However, the methodology adopted to evaluate the closure was based on the superimposition of photos acquired at the different control times and, therefore, differs from the one adopted in the present study.

To date, different studies have analyzed the effect of HA in the postextractive healing of healthy patients. Ibrhaeem et al. [42] compared HA, applied both as a gel and a spray, with no treatment in the healing of extraction sockets, and found a beneficial effect in favor of HA in the immediate postoperative healing. HA gel was seen to offer better results when compared to the spray; however, the difference was not found to be statistically significant. In agreement, Kim JJ et al. [43] investigated the effect of HA in improving wound healing and bone formation after teeth extraction in sockets with chronic pathology in dogs. The authors’ findings showed promising results of HA in enhancing wound healing and subsequential bone formation. On the contrary, the study of Guazzo et al. [44] was found in disagreement with the above-mentioned studies. The Authors investigated the application of amino acid and sodium hyaluronate gel after third molar surgical extraction. The results of the study did not highlight any significant difference in the post-extraction healing between the application of the tested product and the control group (no treatment).

In conclusion, research is continuing to focus on the interaction that systemic conditions may have on the dental treatment of patients [45,46]. The present study reports positive effects of the employment of HA as an adjunct treatment for DM patients who require tooth extractions. If confirmed in further research, the results of the present study may have clinical implications representing a treatment modality that may be routinely applied in the teeth extractive treatments of DB type 2 patients to improve both the quality and the timing of postextractive wound healing. Data about its usage as an adjunct treatment are promising. However, further research is needed to confirm the results and widen the knowledge on the topic, which currently remains a matter of research.

## 5. Conclusions

Based on the clinical results of the present study, HA seems to be promising in improving the timing and the quality of postextractive wound healing in DM type 2 patients. Further clinical research, as well as histological investigations, are required to confirm the results.

## Figures and Tables

**Table 2:** Systemic risk classification.

Low/Absent Systemic Risk	Moderate Systemic Risk	High Systemic Risk
End-organ disease score 0	End-organ disease score ≤ 2	End-organ disease score > 2
Diagnosis ≤ 5 years	Diagnosis between 6 and 10 years	Diagnosis > 10 years
Usual blood sugar levels < 180 mg/dL	Usual blood sugar levels 180–240 mg/dL	Usual blood sugar levels > 240 mg/dL
More than 3 positive parameters between: - no hospitalizations - no episodes of ketoacidosis - no episodes of hypoglycemia - hypoglycemic therapy - controlled diabetes - no changes in therapy	Less than 3 positive parameters between: - no hospitalizations - no episodes of ketoacidosis - no episodes of hypoglycemia - hypoglycemic therapy - controlled diabetes - no changes in therapy	Less than 3 positive parameters between: - no hospitalizations - no episodes of ketoacidosis - no episodes of hypoglycemia - hypoglycemic therapy - controlled diabetes - no changes in therapy

**Table 3:** Subjects’ baseline demographic and clinical characteristics. Data are means (±SD) or percentage (numbers), ^1^ medium glycemic control with the current therapy, ^2^ insufficient glycemic control despite the therapy, ^3^ no glycemic control. HbA1c, Glycated hemoglobin.

	% (n)
Sex	
Male, % (*n*)	47% (17)
Female, % (*n*)	53% (19)
Ethnic origin	
Caucasian	100% (36)
Age (years, mean ± SD)	67.3 ± 11.2
Smokers	25% (9)
Duration of diabetes	11.4 ± 9.9
Body Mass Index (BMI)	28.2 ± 4.8
Preoperative diabetes status:	
<180 mg/dL ^1^	83% (30)
>180 and <240 mg/dL ^2^	17% (6)
>240 mg/dL ^3^	0% (0)
HbA1c (%, mean ± SD)	7.41 ± 1.02
End-organ disease score:	
Cardiomyopathy	86.1% (31)
Nephropathy	19.4% (7)
Peripheral vasculopathy	2.8% (1)
Retinopathy	0% (0)
Systemic risk:	
Low	0% (0)
Medium	86% (31)
High	14% (5)

**Table 4:** Baseline pre-operative data recorded regarding teeth status and surgical difficulty for both T and C groups.

	T Group	C Group
	**Preoperative surgical difficulty**	
Low	80.6%	75%
Medium	19.4%	25%
*p*	0.38	
	**Preoperative crown integrity**	
Intact	58.3%	61.1%
Broken	27.8%	33.3%
*p*	0.47	
	**Root anatomy**	
Low difficutly	80.6%	75%
Medium difficutly	19.4%	25%
*p*	0.38	
	**Pulp vitality**	
Vital	38.9%	30.6%
Necrotic	58.3%	63.9%
Endodontically treated	2.8%	5.6%
*p*	0.67	
	**Presence of cavity**	
Negative	38.9%	33.3%
Positive	61.1%	66.7%
*p*	0.40	
	**Periodontopathic**	
Positive	66.7%	61.1%
*p*	0.40	
	**Precence of radriographically visible periapical osteolysis**	
Negative	94.4%	94.4%
Positive	5.6%	5.6%
*p*	0.9999	

**Table 5:** Data related to the healing index recorded at different time points. Statistical significant results are highlighted with *.

Healing Index	D3		D7		D14		D21	
	**T**	**C**	**T**	C	T	C	T	C
**Mean**	6.6	7.6	5.0	5.9	4.1	4.5	4.0	4.0
**Standard deviation**	1.8	1.9	1.2	1.7	0.5	1.0	0.0	0.0
***p***	0.08		0.01 *		0.02 *		1	
**% of Excellent healing (score n. 4)**	5.6 (*n* = 2)	0 (*n* = 0)	44.4 (*n* = 16)	30.06 (*n* = 11)	97.2 (*n* = 35)	72.2 (*n* = 26)	100 (*n* = 36)	100 (*n* = 36)
***p***	0.23		0.16		0.004 *		1	

**Table 6:** Values recorded regarding socket closure at different time points. Statistical significant results are highlighted with *.

Socket Closure	D0		D3		D7		D14		D21	
**Group**	**T**	**C**	**T**	**C**	T	C	T	C	T	C
**Mean**	148.44	145.74	0.25	0.45	0.05	0.14	0.00	0.02	0.00	0.00
**Standard deviation**	125.38	105.59	0.22	0.29	0.06	0.17	0.01	0.04	0.00	0.00
***p***	0.30		0.04 *		0.001 *		0.001 *		1	
**% of socket fully closed (n)**	/	/	0	0	27.78 (*n*= 10)	33.33 (*n* = 12)	77.78 (*n* = 28)	61.11 (*n* = 22)	100 (*n* = 36)	100 (*n* = 36)
***p***	/		/		0.8		0.57		0.33	

**Table 7:** Data recorded regarding VAS scale at different time points.

VAS	D3		D7		D14		D21	
	**T**	**C**	**T**	C	T	C	T	C
Mean	3.63	5.38	2.23	3.08	0.15	0.38	0.17	0.17
Standard deviation	1.77	2.39	1.81	1.59	0.38	0.51	0.39	0.39
*p*	<0.001		0.04		0.03		0.9999999	

## Data Availability

All data and materials are available from the corresponding author upon reasonable request.
